# Contextual Fear Memory Formation and Destabilization Induce Hippocampal RyR2 Calcium Channel Upregulation

**DOI:** 10.1155/2018/5056181

**Published:** 2018-07-05

**Authors:** Jamileth More, María Mercedes Casas, Gina Sánchez, Cecilia Hidalgo, Paola Haeger

**Affiliations:** ^1^Biomedical Neuroscience Institute, Faculty of Medicine, Universidad de Chile, Santiago, Chile; ^2^Pathophysiology Program, ICBM, Faculty of Medicine, Universidad de Chile, Santiago, Chile; ^3^Center for Exercise, Metabolism and Cancer, Faculty of Medicine, Universidad de Chile, Santiago, Chile; ^4^Department of Neurosciences and Physiology and Biophysics Program, ICBM, Faculty of Medicine, Universidad de Chile, Santiago, Chile; ^5^Department of Biomedical Sciences, Faculty of Medicine, Universidad Católica del Norte, Coquimbo, Chile

## Abstract

Hippocampus-dependent spatial and aversive memory processes entail Ca^2+^ signals generated by ryanodine receptor (RyR) Ca^2+^ channels residing in the endoplasmic reticulum membrane. Rodents exposed to different spatial memory tasks exhibit significant hippocampal RyR upregulation. Contextual fear conditioning generates robust hippocampal memories through an associative learning process, but the effects of contextual fear memory acquisition, consolidation, or extinction on hippocampal RyR protein levels remain unreported. Accordingly, here we investigated if exposure of male rats to contextual fear protocols, or subsequent exposure to memory destabilization protocols, modified the hippocampal content of type-2 RyR (RyR2) channels, the predominant hippocampal RyR isoforms that hold key roles in synaptic plasticity and spatial memory processes. We found that contextual memory retention caused a transient increase in hippocampal RyR2 protein levels, determined 5 h after exposure to the conditioning protocol; this increase vanished 29 h after training. Context reexposure 24 h after training, for 3, 15, or 30 min without the aversive stimulus, decreased fear memory and increased RyR2 protein levels, determined 5 h after reexposure. We propose that both fear consolidation and extinction memories induce RyR2 protein upregulation in order to generate the intracellular Ca^2+^ signals required for these distinct memory processes.

## 1. Introduction

Fear conditioning, an associative learning process that produces robust memories, represents a form of Pavlovian conditioning that has received considerable attention over the last years [1, 2]. When exposed to fear conditioning protocols, animals acquire fear memory through the association between conditioned stimuli—a tone, a smell, or a context—with an unconditioned aversive stimulus, usually a foot shock. Evidence that the hippocampus forms part of the neuronal pathways involved in contextual fear conditioning came first from studies showing that lesions in the dorsal hippocampus prevent both the acquisition and the expression of context-dependent fear conditioning [3–5]. Recent optogenetic techniques support hippocampal involvement in context-dependent fear conditioning [6]. Other reports indicate that synaptic plasticity in the amygdala mediates the association between conditioned and unconditioned stimuli, whereas hippocampal synaptic plasticity mediates contextual coding [2, 7–9].

In agreement with the procedures developed by Pavlov many years ago [10], protocols to study memory destabilization entail special procedures, whereby animals previously exposed to fear conditioning protocols are reexposed subsequently to the conditioned stimulus in the absence of the unconditioned aversive stimulus. This procedure activates memory retrieval, a dynamic phenomenon that depending on the length of the reexposure session triggers two distinct processes, reconsolidation or extinction. Brief reexposition triggers a labile state that requires de novo protein synthesis to restabilize memory persistence in a process known as reconsolidation [11]. In contrast, prolonged, nonreinforced retrieval sessions induce memory extinction [12]. During the formation of extinction memory, a new learning process occurs, which interferes with the expression of the original memory [13–17]. In addition, it has been proposed that extinction does not destroy or erase the original association between conditioned and unconditioned stimuli, so that the expression of extinction memory represents the formation of a new memory that depends on the context [18]. Studies performed in rodents revealed that the dorsal hippocampus is involved in the acquisition, contextual encoding, and context-dependent retrieval of fear memory extinction [13]. Subsequent reports revealed that prefrontal modulation of amygdala activity mediates the context specificity of the extinction process and that the hippocampus has a fundamental role in contextual memory retrieval [19]. Furthermore, CA1 infusion with the GABA_A_ agonist muscimol before the extinction session impaired extinction, showing that the hippocampal CA1 region plays an important role in the fear extinction process [20].

Neuronal Ca^2+^ signals play key roles in memory processes [21], including fear memory [22]. Activity-generated neuronal Ca^2+^ signals arise from Ca^2+^ influx mediated by N-methyl-D-aspartate receptors (NMDAR) and voltage-gated Ca^2+^ channels (VGCC). Calcium release from the endoplasmic reticulum (ER) mediated by inositol 1,4,5-trisphosphate receptor (IP_3_R) and ryanodine receptor (RyR) channels also contributes to generate Ca^2+^ signals in response to neuronal activation [23–25]. In a rodent brain, immunohistological techniques have revealed the heterogeneous expression pattern of both receptor types within neuronal cells [26, 27]. Mammals express three RyR isoforms; specific genes, identified and cloned, encode each isoform [28]. The brain expresses all three RyR isoforms; of these, the RyR2 isoform is the predominant isoform expressed in rat and chicken brain [29, 30]. As detailed below, the redox-sensitive RyR2 isoforms have key roles in hippocampal structural plasticity and spatial memory processes [31].

The generation of intracellular Ca^2+^ signals promotes RyR activity, giving rise to a cellular response known as Ca^2+^-induced Ca^2+^ release (CICR). In neuronal cells, Ca^2+^ influx mediated by NMDAR and VGCC elicits RyR-mediated CICR, which operates as an amplification mechanism of postsynaptic Ca^2+^ entry signals [32]. The resulting amplification and propagation of the initial Ca^2+^ entry signals are presumably a necessary event for the induction of synaptic plasticity and for activity-induced gene expression in hippocampal neurons [32–37]. Hippocampal neurons possess in their soma, axons, dendrites, and dendritic spines the structural and molecular machinery that underlies CICR [33, 38, 39]. In effect, the ER forms an intricate continuous network in neuronal cells that is present in the soma and extends towards the axons, dendrites, and dendritic spines [40, 41]. Treatment of primary hippocampal neurons with the RyR agonist caffeine or with brain-derived neurotrophic factor (BDNF) promotes RyR-dependent dendritic spine remodeling, leading to increased density and length of dendritic spines [38, 42, 43]. In addition, RyR2 downregulation abolishes BDNF-induced spine remodeling in primary hippocampal neurons [31]. These findings indicate that the RyR2 isoform plays a key role in hippocampal structural plasticity.

Calcium release mediated by RyR channels is becoming an important subject in the study of learning and memory under normal and pathological conditions. Several studies employing different paradigms or conditioning tasks have described that RyR-mediated Ca^2+^ release plays a central role in the acquisition and/or consolidation of memory processes [24, 25]. Training rats in the Morris water maze, a classical spatial memory task, increases hippocampal RyR2 protein levels and mRNA expression 12 and 24 h after training [44], suggesting RyR2 involvement in spatial memory processes. Rats trained in the Morris water maze also display significant RyR2 and RyR3 upregulation at the fifth day of training, and these changes persist until the memory consolidation phase (ninth day) [43]. In addition, successful long-term performance of a hippocampus-dependent spatial task (object location) increases the hippocampal protein levels of RyR2, RyR3, and IP_3_R type-1 (IP_3_R1) Ca^2+^ channels [45]. Other studies have shown that in a learning model, in which chickens were trained in a passive avoidance discrimination task, RyR channel inhibition with dantrolene (administered immediately after training) causes loss of memory retention [30]. In contrast, the RyR agonist 4-chloro-m-cresol (4-CMC) administered immediately after training chickens in a passive discrimination avoidance task results in high memory retention that persists for up to 24 h after training, an indication of enhanced memory consolidation [46]. In mice, RyR channel inhibition hampers memory retention in animals conditioned in inhibitory avoidance or radial arm-maze tasks [29, 47], while studies involving administration to mice of antisense oligodeoxynucleotides directed at each RyR isoform indicate that selective knockdown of RyR2 and RyR3, but not of RyR1, impairs memory retention in a passive avoidance test [29]. Highlighting the key role of the RyR2 isoform in memory processes, a recent study performed in rats showed that RyR2 downregulation by intrahippocampal injection of RyR2-directed antisense oligodeoxynucleotides causes conspicuous defects in a previously memorized spatial memory task [31]. To our knowledge, however, information is lacking regarding whether fear memory formation, consolidation, or extinction entail RyR channel function or expression.

In this work, we measured RyR2 protein content in the hippocampus isolated from rats exposed to context-dependent fear conditioning or to subsequent retrieval sessions aimed at destabilizing fear memory by triggering extinction memory. We found that both consolidation and destabilization of contextual fear conditioning resulted in significant increases of RyR2 protein content in the rat hippocampus.

## 2. Methods

### 2.1. Experimental Animals

Sprague-Dawley rats (males, 2.5-month average age) weighing 230–250 grams were used in this study. Animals housed in suitable cages (3 animals per cage) were maintained with a light/dark cycle of 12 h, at an average temperature of 22°C with food and water ad libitum. The animals were handled (habituation to the environment) 2 days before the initiation of conditioning protocols. All experimental protocols used in this work complied with the “Guiding Principles for Research Involving Animals and Human Beings” of the American Physiological Society and were approved by the Bioethics Committee on Animal Research, Faculty of Medicine, Universidad de Chile.

### 2.2. Context-Dependent Fear Conditioning

The animals were trained and tested in a conditioning chamber (Startle and Fear Conditioning System, PANLAB, Barcelona, Spain), equipped with stainless steel bars in the floor through which the animals received electrical stimulation. The “Freezing Software” program (PANLAB) was used to analyze the behavioral response known as freezing, defined as the complete absence of movement except breathing. The study engaged four different experimental groups: (1) control rats (C), which were exposed to the context but did not receive electrical stimulation; (2) rats exposed to the context unpaired with electrical stimulation (US); (3) trained rats (T5 and T29), which were exposed to the context paired with electrical stimulation, and (4) reexposed rats, which after exposure to the context paired with electrical stimulation, were reexposed 24 h later to the context without the aversive stimulus for 3 min (R3), 15 min (R15), or 30 min (R30).

Each group of rats underwent separately some of the following sessions (see Supplementary Figure 1 for a complete description of the experimental protocols employed). *Habituation session*: one day before exposure to the fear conditioning protocol, all animals were habituated to the conditioning chamber for a period of 3 min; the freezing behavior displayed in this habituation session was used to set up the equipment. After this initial exposure session without aversive electrical stimulus, all rats were returned to their respective cages. *Training sessions*: the animals were exposed 24 h after the habituation session to the conditioning chamber for 5 min. In this period, the animals of the control (C) group did not receive an aversive stimulus. The animals of the T5, T29, and the three R groups received two sets of paired electrical stimuli: the first stimulus (0.7 mA, for 2 s) was applied two min and the second stimulus (0.7 mA, for 2 s) four min after the rats entered the training chamber. The session concluded with a recovery time of 58 seconds; after this lapse, the animals were returned to their respective cages. The rats of the US group received only one unpaired electrical stimulus (0.7 mA, for 4 s) as soon as they entered the conditioning chamber and were removed immediately to their cages. *Reexposure sessions*: the animals were reexposed 24 h after the training session for 3 min (R3), 15 min (R15), or 30 min (R30) to the conditioning context, without the aversive stimulus. *Test sessions*: all test sessions comprised exposure to the conditioning chamber without electric stimulus, as detailed below. In the test period, motor activity was measured continuously using the “Freezing Software” program. The freezing behavior of rats of the T5 and US groups was tested 5 h after training, while the rats of the T29 group were tested 29 h after training; in all cases, this last test session comprised 5 min of exposure to the context without the aversive stimulus. All the rats of the reexposed groups were tested 24 h after training, as detailed next. The freezing behavior of rats from the R3 group was measured during the 3 min period of reexposure and 5 h later. The freezing behavior of rats of the R15 and R30 groups was measured during the first 5 min of reexposure, during the entire duration of the respective reexposure session, and 5 h later in a test session that comprised 5 min of exposure to the context without the aversive stimulus. In all cases, the rats underwent euthanasia right after the last test session, and the hippocampus was removed for RyR2 protein determination.

### 2.3. Western Blot Analysis for RyR2 Protein Detection

The isolated hippocampus was homogenized with a glass/Teflon homogenizer in 200 *μ*l of lysis solution (20 mM BAPTA; 10 mM MOPS-Tris, pH 7.5; leupeptin 100 *μ*g/ml; and pepstatin 50 *μ*g/ml). The resulting suspension was incubated on ice for 10 min, sonicated 3 times (20 s each time) and centrifuged at 3000 ×g for 25 min at 4°C. The supernatant was mixed with 1% NP40, shaken until dissolved (total extract), and aliquots were stored at −80°C. Protein concentration was measured with the sulfosalicylic acid Protein Assay Kit (Thermo Scientific, Rockford, IL, USA). For Western blot analysis, the above total extracts were denatured with 4x reducing buffer (34.8% glycerol, 1 M Tris base, 2 mM EDTA, 0.1 M dithiothreitol (DTT), 8% SDS, and 0.4% bromophenol blue). Electrophoresis was performed in 3.5–8% discontinuous gradient polyacrylamide gels, containing a 15% stacking layer to favor the separation of the different RyR isoforms without losing the *β*-actin band. Gels were immersed in Tris-Tricine buffer (6 mM Tricine, 1 mM EDTA, and 12.5 mM Bis-Tris propane, pH 8.0) and run for 4 h at 80 V. Next, protein bands were transferred (350 mA, 2.5 h) to PVDF membranes (Millipore Corp., Bedford, MA, USA) using the Transfer-Blot R Turbo System (Bio-Rad, Hercules, CA, USA) and Tris-Tricine transfer buffer with 10% methanol. PVDF membranes were incubated overnight at 4°C with blocking buffer containing 5% milk and were then incubated under constant stirring at room temperature for 2 h in 5% milk with specific antibodies against RyR2 (Anti-RyR2, Thermo, Waltham, CA, USA; 1 : 1000) or against *β*-actin used as loading control (Anti-*β*-actin, Sigma, San Luis, MI, USA; 1 : 12000). Membranes were washed next with 2% Tween in Tris-buffered saline (TBS, 3 washes, 10 min each) and were then incubated with conjugated secondary anti-mouse antibodies (Cell Signaling, Danvers, MA). The membranes were visualized with a chemiluminescence system (Amersham Biosciences, Piscataway, NJ, USA). Films were scanned and analyzed with the ImageJ software.

### 2.4. Immunofluorescence

Adult rats were perfused transcardially with 4% paraformaldehyde (Sigma, St. Louis, MI). The rats of the T5 fear-trained group, plus their respective untrained controls, were perfused 5 h after the 5 min training session. The rats of the reexposed R15 group were perfused 5 h after the 15 min reexposure session, while rats belonging to the T29 group were perfused right after the 5 min test session performed 29 h after the training session. As controls, naïve rats of the same age and weight were perfused as above. After perfusion, the brains were removed and placed in 4% paraformaldehyde for 2 h. The brains were incubated next for 72 h in a solution containing 30% sucrose, 0.001% sodium azide. Slices (30 *μ*m) were cut with a microtome at −30°C. Free-floating sections were bathed in phosphate-based saline (PBS) buffer (mM: 137 NaCl, 2.7 KCl, 10 Na_2_HPO_4_, and 1.2 K_2_HPO_4_), containing 0.25% Triton X-100 (PBS-TX) plus 3% donkey serum, for 2 h at room temperature, and were incubated overnight at 4°C with PBS-TX containing RyR2 antibody (1 : 50, Thermo, Waltham, CA, USA). Sections were washed for 5 min in PBS and were incubated next for 2 h with Alexa Fluor 488 anti-mouse antibodies (1 : 300, Thermo, Waltham, CA, USA). Brain tissue slices, washed in PBS, were mounted on glass slides and covered with mounting medium. DAPI (Sigma, St. Louis, MI, USA) was employed for nuclear staining. Slices from −3.3 mm of the bregma [48] were chosen to analyze the expression of RyR2 in the CA1, CA3, and dentate gyrus (DG) hippocampal regions; the dorsal third and lateral ventricles were taken as place references in the slices. A z-image stack of 1.5 *μ*m sections was captured from the CA1, CA3, and DG regions using a confocal microscope (Nikon C2+). Fluorescence intensity was measured using the NIS-Elements software viewer 4.0 and ImageJ free viewer software.

### 2.5. Statistical Analysis

Results are expressed as mean ± SE. Statistical significance was evaluated with the GraphPad Prism 5 software. To test for statistical significance, unpaired or paired Student's *t*-test and one-way ANOVA followed by Tukey's multiple comparison post hoc test or repeated measures ANOVA were used, as detailed in the figure legends.

## 3. Results

### 3.1. Contextual Fear Conditioning Caused a Transient Increment of RyR2 Protein Content

**Figure 1:**
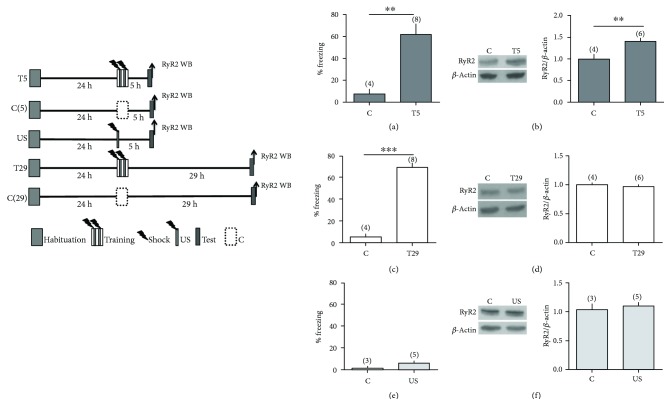
Context conditioned fear memory consolidation causes transient increases in RyR2 protein content. (a, c) Memory retention (% freezing) was measured 5 h (T5) or (c) 29 h (T29) after training or after context exposure without the aversive stimulus as control. (b, d) RyR2 protein content was analyzed 5 and 29 h after training, respectively. Representative blots and bar graphs illustrate RyR2 hippocampal content in each group of rats. (e, f) Memory retention and RyR2 hippocampal protein contents were measured (c) 5 h after exposure to the context or after unpaired stimulation (US). Values represent mean ± SE; the number of independent determinations is indicated in each graph. Statistical analysis was performed with unpaired Student's *t*-test; ^∗∗^ *p* < 0.01, ^∗∗∗^ *p* < 0.001.

Male rats exposed to the contextual fear conditioning protocol were tested 5 h or 29 h after the training session. In this test session, animals spent 5 min in the conditioning chamber in the absence of the aversive stimulus. Immediately after this test session, the rats underwent euthanasia and the hippocampus was isolated for Western blot (WB) analysis (Figure 1, left panel). The animals tested 5 h after training (T5) showed a significant (~7-fold) increase in freezing behavior compared to controls (Figure 1(a)), a clear-cut indication of fear memory retention.

**Figure 2:**
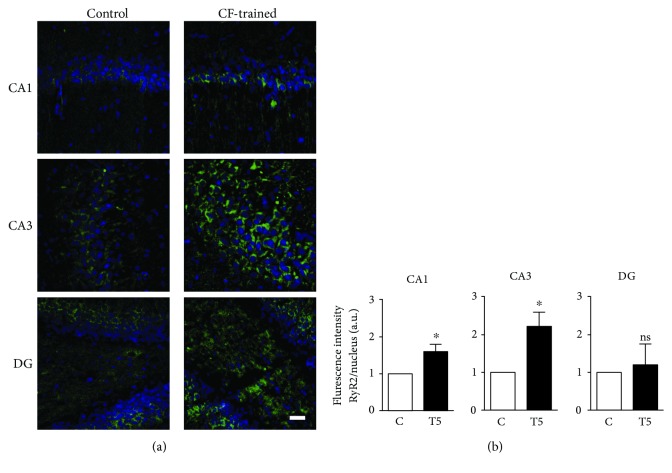
Contextual fear training promotes RyR2 upregulation in situ. (a) Representative confocal images of RyR2 immunofluorescence (green) and nuclear stain (blue) obtained from the CA1, CA3, and DG hippocampal regions. Samples were obtained 5 h after training animals (T5) in the contextual fear protocol or from controls similarly exposed to the context in the absence of the electric shock. Scale bar: 20 *μ*m. (b) Normalized RyR2 immunofluorescence values. To calculate these values, RyR2 immunofluorescence in the entire image was divided in each case by the corresponding nuclear stain, and the control ratios were set as 1. Values represent mean ± SE; *N* = 3 for the T5 and the control groups. Statistical analysis was performed with paired one-tailed Student's *t*-test. ^∗^ *p* < 0.05; ns: not significant.

The increased freezing behavior persisted in the group of animals tested 29 h after the training section (T29) (Figure 1(c)), indicating fear memory consolidation. Densitometry analysis of blots from hippocampal samples isolated 5 h after the training session (T5) revealed a significant increase (~1.5-fold) in RyR2 protein content compared to controls (Figure 1(b)), indicating that the RyR2 protein increase was due to contextual fear training and not to exposure to the context. This RyR2 increment was transient; 29 h after the training session, the hippocampal RyR2 protein levels in animals of the T29 group did not differ significantly from the levels displayed by the controls (Figure 1(d)). Additional analysis by immunohistochemistry of hippocampal sections isolated 5 h after the training session of the T5 group rats (see training scheme in Figure 1) showed that contextual fear (CF) training incremented RyR2 protein immunostaining (green) in the CA1 and CA3 hippocampal regions relative to their respective controls, as illustrated in Figure 2.

To assess if the RyR2 protein increase was due to an association between the electric shock and the context or occurred by an unspecific effect of the shock itself, we tested a separate group of animals, the US group. To this aim, rats from the US group received an electric shock (0.7 mA for 4 s) as soon as they entered the chamber and were removed immediately to their cages (Figure 1, left panel). As illustrated in Figure 1(e), this protocol did not generate associative learning in response to the context, as evidenced by the low freezing behavior exhibited by the US animals tested after 5 h, and did not result in increased RyR2 protein levels (Figure 1(f)). We interpret these combined findings as an indication that the RyR2 increase displayed by fear-trained animals 5 h after the training session stemmed from the associative learning induced by exposure to the contextual fear protocol.

### 3.2. Conditioned Fear Extinction Increases Hippocampal RyR2 Protein Levels

**Figure 3:**
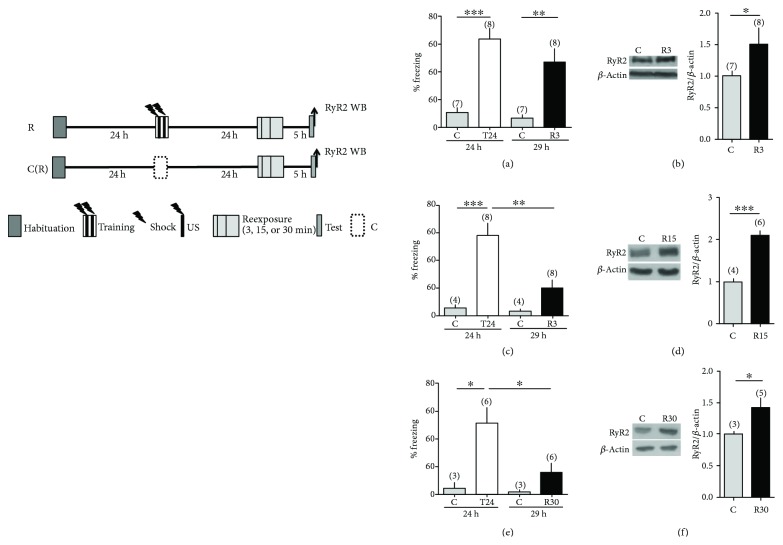
Context reexposure for 3, 15, or 30 min induces memory extinction and enhances RyR2 expression. (a, c, and e) Memory retention was evaluated 24 h after training (T24) or 5 h after reexposure to the context for 3 min (R3), 15 min (R15), or 30 min (R30). Control rats did not receive electrical stimulation during the training session and were evaluated 5 h after reexposure to the context for 3 min, 15 min, or 30 min. (b, d, and f) RyR2 expression was analyzed 5 h after performing the respective reexposure sessions (see scheme at left). Representative blots and quantification (bar graphs) showing hippocampal RyR2 protein content in each group of rats. Values represent mean ± SE. The number of independent determinations is indicated in each graph. Statistical analysis in (a, c, and e) was done with one-way ANOVA followed by Tukey's multiple comparison post hoc test; statistical analysis in (b, d, and f) was performed with Student's *t*-test. ^∗^ *p* < 0.05, ^∗∗^ *p* < 0.01, ^∗∗∗^ *p* < 0.001.

To study whether an increase in hippocampal RyR2 protein content also occurred after exposure to retrieval sessions that promote memory destabilization, 24 h after the training session, the rats were reexposed for 3 min (R3), 15 min (R15), or 30 min (R30) to the conditioning context without the aversive stimulus. Control animals were reexposed for these same times (Figure 3, left panel). The freezing behavior of animals belonging to the R3 group was evaluated during the 3 min reexposure session; freezing in animals of the R15 and R30 groups was measured during the initial 5 min period and during the entire duration of the respective reexposure sessions. An additional 5 min test session was performed 5 h later for all animals. Immediately after this last test session, rats underwent euthanasia and the hippocampus was removed for RyR2 protein determination (Figure 3, left panel). Control animals presented under all situations low freezing behaviors during the respective reexposure sessions and the test sessions performed 5 h later (Figures 3(a), 3(c), and 3(e)). The animals reexposed for 3 min to the context without the aversive stimulus (R3) displayed in this period significant fear-associated memory retention, with prominent freezing behavior (63.6 ± 7.6%). Yet, when tested 5 h later, these animals displayed significantly lower freezing (47.2 ± 9.5%), as illustrated in Supplementary Figure 2B, and presented a significant increase (1.85 ± 0.23; *N* = 6) in RyR2 protein content (Figure 3(b)). In contrast, RyR2 protein content did not increase in the controls reexposed (24 h after the first exposure session) to the context for 3 min without a shock and tested 5 h later (Figure 3(b)).

As an additional control, the RyR2 protein content was determined after the 3 min reexposure session (T24–3, Supplementary Figure 2A); no changes in RyR2 protein levels relative to the control were observed in these conditions. Accordingly, we conclude that the RyR2 protein content increase induced by the 3 min reexposure session did not happen immediately after the session but took place a few hours postreexposure. We propose (see “Discussion”) that early extinction-dependent mechanisms mediate the RyR2 upregulation induced by this short reexposure session.

Animals reexposed to the context for 15 min (R15) exhibited a significant decrease in freezing behavior during the reexposure session, from 57.0 ± 9.0% during the first 5 min of reexposure to 35.0 ± 6.7% determined during the entire 15 min session (Supplementary Figure 2C). When tested 5 h later (Figure 3(c)), these same animals showed an even larger decrease in freezing (19.9 ± 6.3%), which was accompanied by a sizeable RyR2 protein increase (Figure 3(d)).

Likewise, the animals reexposed for 30 min (R30) displayed a significant decrease in freezing behavior during the reexposure session, from 51.1 ± 11.0% freezing when measured during the first 5 min of reexposure to 21.0 ± 4.0% freezing when measured during the entire length of the reexposure session (Supplementary Figure 2D). When tested 5 h later, these rats displayed freezing behavior with values of 16.0 ± 6.4% (Figure 3(e)) and exhibited a significant increase in RyR2 protein content (Figure 3(f)).

Based on these combined results, we conclude that prolonging the retrieval session to 15 or 30 min without the aversive stimulus results in improved extinction of the previously acquired memory in comparison with reexposure for only 3 min (Supplementary Figures 2E and 2F). The extinction process was even more evident in animals tested 5 h after the reexposure sessions and was accompanied by significant RyR2 protein increases. The RyR2 increase peaked 15 min after reexposure to the context and did not increase further after the 30 min reexposure session (Figures 3(d) and 3(f)). In contrast, control rats reexposed to the context for 15 or 30 min without previous fear memory training did not present RyR2 upregulation (Figures 3(d) and 3(f)); we interpret these findings as an indication that fear memory extinction caused this increase.

### 3.3. Conditioned Fear Extinction Increases RyR2 Immunofluorescence in the Hippocampal CA1, CA3, and DG Regions

We used immunofluorescence assays of fixed brain samples to detect RyR2 protein levels in the different hippocampal regions. Samples from rats (R15) reexposed to the context for 15 min (24 h after training) and tested 5 h later were collected for RyR2 fluorescence immunodetection assays. Their immunofluorescence patterns were compared with those displayed by naïve rats or by rats (T29) trained in the contextual fear conditioning protocol, which exhibited robust memory in the test session performed 29 h later (Figure 1(c)) but presented no changes in hippocampal RyR2 protein content in immunoblots (Figure 1(d)).

**Figure 4:**
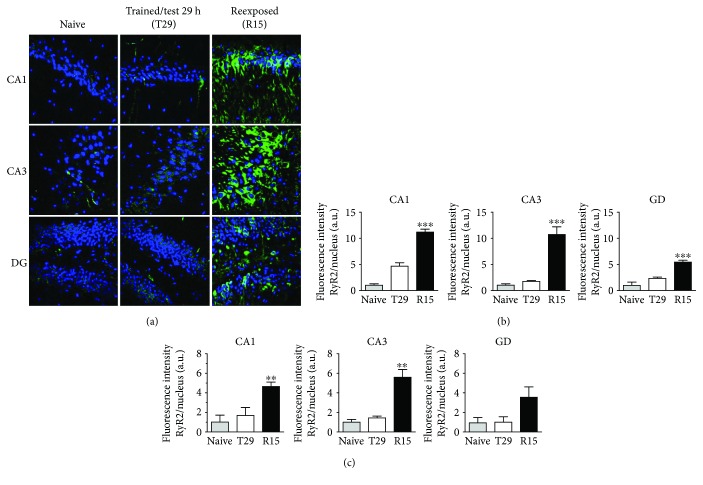
Memory extinction promotes RyR2 upregulation in situ. (a) Representative confocal images of RyR2 immunofluorescence (green) and Hoechst nucleus stain (blue) obtained from CA1, CA3, and DG region of the hippocampus. Samples were obtained 29 h after training (T29) or 5 h after the 15 min reexposure session (R15). Samples from naïve rats were used as control. Scale bar: 20 *μ*m. (b) Bar graphs showing the quantification of RyR2 immunofluorescence pixels normalized by nuclear stain present in regions enriched in nuclei in CA1, CA3, and DG. (c) Bar graphs showing the quantification of RyR2 immunofluorescence pixels, normalized by the nuclear stain present in the entire images of CA1, CA3, and DG. Values represent mean ± SE; *N* = 3 for samples from the control and T29 groups; *N* = 4 for the R15 group. Statistical analysis was done with one-way ANOVA (CA1 *p* = 0.0001, CA3 *p* = 0.0008, and DG *p* = 0.0009) followed by Tukey's multiple comparison post hoc test; ^∗∗^ *p* < 0.01; ^∗∗∗^ *p* < 0.001 with respect to naïve and T29, respectively.

The rats belonging to the R15 and T29 groups were perfused immediately after the last test sessions; in all cases, the aversive stimulus was applied only in the training sessions.  Figure 4(a) illustrates RyR2 immunofluorescence staining (green) and nuclear staining (blue) in the hippocampal CA1, CA3, and DG regions. Although the naïve and T29 groups displayed some weak Ry2 protein staining, hippocampal RyR2 immunofluorescence was higher in sections from the R15 group.

The graph presented in Figure 4(b) illustrates the quantification of the immunofluorescence images acquired from regions enriched in nuclei; the graph presented in Figure 4(c) illustrates the quantification of the immunofluorescence acquired from the complete images. All values represent the ratio between RyR2 immunofluorescence and nuclear stain. We conclude from these results that the 15 min reexposure session induced significant increments in RyR2 immunofluorescence in the CA1 and CA3 regions, which was particularly evident in regions enriched in nuclei (Figure 4(b)). The RyR2 immunofluorescence increase displayed by the DG region was less prominent when evaluated in nuclei-enriched regions (Figure 4(b)), but this increase did not reach statistical significance when evaluated in the entire image (Figure 4(c)).

Although a direct comparison between RyR2 immunoblot determinations (performed in whole hippocampus homogenates) and immunofluorescence images is not accurate, quantification of the whole immunofluorescence images (Figure 4(c)) yielded values closer to those presented in the immunoblots illustrated in Figure 3(d). In contrast, samples from rats tested 29 h after training, which did not undergo reexposure to the context (Supplementary Figure 1), displayed similar RyR2 immunofluorescence images as those exhibited by naïve rats (Figure 4(a); for details, see Supplementary Figure 3). Quantification of T29 RyR2 immunofluorescence images yielded values not significantly different from those displayed by the corresponding regions from naïve rats (Figures 4(b) and 4(c)). These results are in agreement with the immunoblot results (Figure 1(d)), which illustrate the lack of RyR2 protein increase displayed by the whole rat hippocampus isolated 29 h after contextual fear training.

## 4. Discussion

Based on the results shown in this work, we suggest that our model of context-conditioned fear memory, a task dependent on the hippocampus [9, 49], effectively promoted learning and memory acquisition, which became consolidated as indicated by the high percentage of freezing in animals evaluated 5, 24, or 29 h after training. Our novel results also show that a transient increase in RyR2 protein levels occurred during consolidation of fear-conditioned memory, since hippocampal RyR2 protein upregulation occurred 5 h posttraining but not 29 h after training. We suggest, accordingly, that the formation of long-term memory, or the early stages of the consolidation phase of fear-conditioned memory, requires transitory RyR2 upregulation in the whole hippocampus in order to generate the Ca^2+^ signals required for fear memory consolidation. Previous reports—showing increased RyR2 upregulation in animals exposed to different hippocampal-dependent memory protocols [31, 43–45]—support our proposal. These combined results place calcium release mediated by RyR2 channels and presumably RyR2 upregulation as well as important events in hippocampal-dependent memory processes.

Using different strategies and memory tasks, several authors have reported that protein synthesis is temporally required to elaborate long-term memory. Specifically, 1 h is the critical period for protein synthesis after contextual fear conditioning training [50]. Additionally, hippocampal inhibition of protein synthesis with anisomycin impairs memory formation when given either 15 min before training or 3 h posttraining in a one trial of inhibitory avoidance training task [51]. Furthermore, the expression of the immediate early genes (IEG) Zif268, c-Fos, and Arc peaks 90 min after the last training trial in the Morris water maze training [52] or in fear memory training [53]. In similarity to the current results, we described previously that the hippocampal RyR2 protein content increased 5 h after training rats in the Morris water maze [43]. Yet, the possibility of an even earlier RyR2 peak remains a subject of future studies, as does the analysis of a possible causal relationship between IEG and RyR2 expression in fear memory formation. A recent report described an important role of protein degradation in memory formation [54], but whether a decrease in RyR2 degradation underlies the transient RyR2 upregulation induced by fear memory training remains to be explored in future studies.

Moreover, when studying the process of conditioned fear extinction, we found that reexposure to the context for 3, 15, or 30 minutes without reinforcement of the electrical stimulus generated a decrease in the conditioned fear response and most likely promoted learning-associated fear memory extinction, as described in previous studies [14–16]. As all learning processes, extinction has three different phases: acquisition, consolidation, and retrieval [55]. In our experimental design, trained animals displayed decreased freezing with increasing times of reexposure to the context; freezing decreased more after reexposure for 15 or 30 min than after 3 min of reexposure, indicating increased formation of extinction memory with time. This freezing decrease was more evident at the test session performed 5 h after reexposure, an indication of more effective extinction of fear-conditioned memory.

Both extinction of the learned response and original learning require acquisition of new information and protein synthesis, which promotes the consolidation of the new information [56]. The present results show that the 3, 15, and 30 min reexposure sessions resulted in marked extinction of fear memory and increased RyR2 protein levels in the whole hippocampus, determined by immunoblot assays. Moreover, reexposure for 15 min produced significant increments of RyR2 immunofluorescence in the CA1, CA3, and DG hippocampal regions, and these changes were especially evident in the regions enriched in nuclei. We suggest, therefore, that the hippocampal RyR2 protein increase forms part of the process of extinction memory generation related to contextual fear. In view of the current results showing the effects of the reexposure session on extinction memory generation, we evaluated the consequences of suppressing this session on RyR2 protein levels in different hippocampal regions. We found that the absence of the reexposure sessions generated high freezing behavior in the test session performed 29 h after training, indicating lack of extinction and significant retention of the original fear-associated memory. Nevertheless, the RyR2 upregulation induced by fear memory extinction did not occur in this case, a clear indication that the extinction process mediates the observed RyR2 protein increase.

Altogether, based on the present results, we suggest that RyR2 upregulation forms part of the processes underlying both fear-associated memory consolidation, which requires a transient increase in RyR2 protein content, and fear memory extinction, which implies the generation of new learning. Different spatial memory protocols induce RyR2 channel upregulation [43–45]. We now add to this list the formation as well as the destabilization of contextual fear memory. The molecular mechanisms underlying RyR2 upregulation in memory formation and extinction remain unreported. We propose that RyR2 upregulation would contribute, via RyR2-mediated CICR, to amplify the Ca^2+^ signals generated by NMDAR or L-type VGCC that are required for hippocampal-dependent fear extinction [57, 58]. The ensuing calcium-dependent signal transduction cascades may engage Scr kinase and CaMKII, both of which have been involved in fear extinction memory [57, 59]. Moreover, contextual fear memory extinction requires activity-dependent expression of BDNF [60], a neurotrophin that induces RyR2 upregulation, as does spatial memory consolidation [43]. Accordingly, RyR2 upregulation may form part of a common mechanism involved in BDNF-mediated memory formation and extinction. According to this view, RyR2 upregulation would ensure the proper amplification and propagation of the initial activity-generated Ca^2+^ signals elicited in postsynaptic neurons by ligand-dependent receptors or L-type VGCC that have been implicated in the learning process [55, 61–63].

A previous study reported that RyR type-3 (RyR3) knockout mice exhibited impairments of performance in contextual fear conditioning, passive avoidance, and Y-maze learning tests [64]. Furthermore, in mice, selective knockdown of RyR2 and RyR3, but not of RyR1, impairs memory retention in a passive avoidance test [29], while training rats in the Morris water maze causes significant RyR2 and RyR3 upregulation at the fifth day of training, and these changes persist until the memory consolidation phase (ninth day) [43]. Here, we present additional findings in wild-type rats showing that contextual fear memory acquisition or extinction upregulates the RyR2 isoform, which when downregulated causes striking spatial memory defects [31]. These combined results highlight the importance of the RyR2/RyR3 isoforms in hippocampal-dependent memory process.

To conclude, it is important to mention that extinction memory is a particularly interesting type of memory, which represents one of the most studied memory forms due to its relevance within the context of some psychiatric disorders such as panic disorders, anxiety, phobias, and posttraumatic stress disorder [65]. Additionally, the extinction memory process is also important for the attenuation of cue-induced drug craving and relapse behavior [66]. Hence, the participation of RyR2 channels as possible key pharmacological targets to these disorders is a relevant subject of future studies.

## Data Availability

All data presented in the article are available upon request.

## Supplementary Materials

Supplementary Materials Complementary information regarding the additional results and protocols used to induce contextual fear memory acquisition and extinction. Supplementary Figure 1: the scheme illustrating the groups of rats exposed to each protocol and the times used to collect the hippocampus for RyR2 determinations. Supplementary Figure 2: results illustrating changes in freezing and RyR2 protein content under additional experimental conditions. Supplementary Figure 3: confocal images of RyR2 immunofluorescence and nuclear DAPI staining, collected in each hippocampal region isolated from different animals.
