# Supplementary material for: Contextual Fear Memory Formation and Destabilization Induce Hippocampal RyR2 Calcium Channel Upregulation
Source: Neural Plast. 2018 Jul 5;2018:5056181. doi: 10.1155/2018/5056181 (PMC6079367; doi:10.1155/2018/5056181)
Supplement: Supplementary Materials — Complementary information regarding the additional results and protocols used to induce contextual fear memory acquisition and extinction. Supplementary Figure 1: the scheme illustrating the groups of rats exposed to each protocol and the times used to collect the hippocampus for RyR2 determinations. Supplementary Figure 2: results illustrating changes in freezing and RyR2 protein content under additional experimental conditions. Supplementary Figure 3: confocal images of RyR2 immunofluorescence and nuclear DAPI staining, collected in each hippocampal region isolated from different animals. [file 5056181.f1.doc]

**Contextual Fear Memory Formation and Destabilization Promote Hippocampal RyR2 Calcium Channel Up-regulation**

Jamileth More^1^, María Mercedes Casas^1^, Gina Sánchez^2,3^, Cecilia Hidalgo^1,3,4^,Paola Haeger^5^

^1^Biomedical Neuroscience Institute, Faculty of Medicine, Universidad de Chile, Santiago, Chile. ^2^Pathophysiology Program, ICBM, Faculty of Medicine, Universidad de Chile, Santiago, Chile. ^3^Center for Exercise, Metabolism and Cancer, Faculty of Medicine, Universidad de Chile, Santiago, Chile. ^4^Department of Neurosciences and Physiology and Biophysics Program, ICBM, Faculty of Medicine, Universidad de Chile, Santiago, Chile. ^5^Department of Biomedical Sciences, Faculty of Medicine, Universidad Católica del Norte, Coquimbo, Chile.

Running title: Conditioned fear memory up-regulates RyR2 channels

Corresponding authors: Paola Haeger and Cecilia Hidalgo

Keywords: Ryanodine, Calcium release, endoplasmic reticulum, synaptic plasticity, memory consolidation, conditioned fear memory.

**Supplementary Figure 1.** Scheme of the protocols used to induce contextual fear memory acquisition and extinction. The scheme also illustrates the times used to collect the hippocampus for RyR2 determination.

**Supplementary Figure 2. A.** Representative immunoblot (left) showing RyR2 protein levels in the hippocampus isolated immediately after context re-exposure for 3 min (T24-3), normalized to its respective control. The graph shown at right illustrates the quantification (N=8), showing that hippocampal RyR2 protein content did not differ from control values when determined right after context re-exposure for 3 min without the aversive stimulus (T24-3), despite the fact that rats re-exposed 24 h after training to the context for 3 min displayed prominent freezing behavior (Figure 2A). **B**. Freezing behavior of rats re-exposed to the context for 3 min (R3), **C** for 15 min (R15) or **D** for 30 min (R30). The freezing behavior of the R3 group (N=8) was tested during the 3 min period of re-exposure (3 min), and in the 5 min test session performed 5 h later (T-5h). The freezing behaviors of the R15 (N=8) and R30 (N=6) groups were tested during the initial 5 min (5 min), during the entire duration of the respective re-exposure sessions (15 min or 30 min), and in the 5 min test session performed 5 h later (T-5h). **E** and **F** compare, respectively, the freezing behaviors of rats from the R3, R15 and R30 groups tested during the sessions (**E**) or 5 h later (**F**). All values represent Mean ± SE. Statistical analysis in **A** was performed with Student’s t-test. **B** For R3 group values, statistical analysis was performed with Student’s t-test; #: p < 0.01. Statistical analysis in **C** (R15), **D** (R30), **E** and **F** was performed with repeated One-way ANOVA (p < 0.0001 for R15, p = 0.006 for R30) followed by Tukey`s Multiple Comparison post-hoc test; *: p < 0.05, **: p < 0.01, ***: p<0.001; ns: not significant.

**Supplementary Figure 3. Memory extinction promotes RyR2 up-regulation *in situ***. Panels **A**), **B**), **C**) show representative confocal pictures of RyR2 immunofluorescence (green) and nucleus stain (blue), taken from the CA1, CA3 and DG hippocampal regions. Samples were obtained 5 h after the 15 min re-exposure session (R15) or 29 h after training (T29). Samples from naïve rats are presented as control. Scale bar: 20 μm.
